# Herbal medicines for the prevention and treatment of cerebral vasospasm after subarachnoid hemorrhage: A protocol for systematic review and meta-analysis

**DOI:** 10.1097/MD.0000000000023388

**Published:** 2020-12-11

**Authors:** Yuna Seo, Han-Gyul Lee, Chul Jin, Seung-bo Yang, Seung-Yeon Cho, Seong-Uk Park, Woo-Sang Jung, Sang-Kwan Moon, Jung-Mi Park, Chang-Nam Ko, Ki-Ho Cho, Seungwon Kwon

**Affiliations:** a Department of Korean Medicine Cardiology and Neurology, Graduate School, Kyung Hee University, Seoul; b Department of Korean Internal Medicine, College of Korean Medicine, Gachon University, Gyeonggi-do; c Department of Cardiology and Neurology, College of Korean Medicine, Kyung Hee University, Seoul, Republic of Korea.

**Keywords:** cerebral vasospasm, herbal medicine, subarachnoid hemorrhage, systematic review

## Abstract

**Background:**

Despite the rapid advances in medical technology, including endovascular interventions and medications, cerebral vasospasm (CVS) after subarachnoid hemorrhage (SAH) is still one of the major threats to the lives of patients with SAH. In East Asian countries, various types of herbal medicines have been used to treat cerebrovascular diseases, including SAH. In this review, we aim to evaluate the efficacy and safety of herbal medicines for the prevention and treatment of CVS after SAH.

**Methods and analysis:**

Seven databases will be searched for relevant studies from inception to the present date “June 2020”. Only randomized controlled trials (RCTs) that assess the effect and safety of herbal medicines for the prevention and treatment of CVS after SAH will be included. The methodological quality will be evaluated using the Cochrane risk of bias assessment tool. After selecting the appropriate studies, a meta-analysis of the RCTs will be performed.

**Results:**

This study will provide a high-quality synthesis of current evidence of herbal medicines for CVS after SAH.

**Conclusion:**

Our systematic review will provide evidence to judge whether herbal medicines are effective interventions for patients with CVS after SAH.

**Ethics and dissemination:**

Ethical approval is not required, as this study is based on a review of published research. This review will be published in a peer-reviewed journal and disseminated electronically and in print.

**Trial registration number:**

Research registry reviewregistry923.

## 1 Introduction

Cerebral vasospasm (CVS) after subarachnoid hemorrhage (SAH) is one of the most common complications, occurring in about 50% of SAH patients, and has been reported to significantly increase disability and mortality.^[1,2]^ The prevalence of symptomatic CVS in delayed ischemic neurological deficit (DIND) has been reported to be 17% to 48%,^[3–5]^ which significantly lowers the quality of life of SAH survivors.^[6]^ DIND could cause secondary cerebral infarction in approximately 50% of cases, resulting in permanent sequelae or death.^[7]^ Therefore, preventing and treating CVS is essential in treating patients with SAH.

According to the current guidelines of the American Stroke Association, the first choice treatment for CVS after SAH is oral administration of nimodipine, a calcium channel blocker.^[8,9]^ In addition, various treatments such as triple H therapy (a combination of hypertension, hypervolemia, and hemodilution), statins, selective inhibitors of phosphodiesterase type-3, and endothelin receptor antagonists have been applied to prevent CVS in patients with SAH.^[10,11]^ However, despite the application of such treatments, CVS in SAH patients is not sufficiently prevented, and the prognosis of SAH survivors with CVS remains at a poor level. For example, several studies have suggested that CVS-associated infarcts occur in 20% to 50% of patients, DINDs in 27% to 37%, and severe morbidity in 30% to 40% of patients with SAH, despite having been treated with nimodipine.^[6,12,13]^

In East Asian countries, various types of herbs have been used to treat cerebrovascular diseases, including SAH. There have already been some clinical and experimental studies on the use of herbal medicines for the prevention and treatment of CVS after SAH. Several herbs, such as *Salvia miltiorrhiza, Acanthopanax senticosus*, *Ginkgo biloba*, *Pueraria lobata*, *Liguisticum chuanxiong*, cow bezoar, *Diospyros kaki*, and *Gynostemma pentaphyllum*, have been reported to be beneficial for the prevention and treatment of CVS.^[14]^ There has also been a study suggesting that the traditional Chinese herbal complex, Qingnao Oral Liquid, could effectively relieve the headache caused by SAH.^[15]^ However, according to our search, there is still no systematic review and meta-analysis evaluating the efficacy and safety of herbal medicine for CVS after SAH. Therefore, we will aim to conduct this systematic review to summarize and assess randomized controlled trials (RCTs) regarding herbal medicines for preventing or treating CVS after SAH.

The aims of this study are as follows:

1. To assess whether adjunct herbal medicine therapies with conventional Western medicine therapies to prevent CVS after SAH are more effective and safer than conventional Western medicine therapies.
2. To assess whether adjunct herbal medicine therapies with conventional Western medicine therapies for the treatment of CVS after SAH are more effective and safer than conventional Western medicine therapies alone.

## 2 Methods

### 2.1 Study registration

The current protocol report adheres to the Preferred Reporting Items for Systematic Reviews and Meta-Analysis Protocol.^[16]^ The protocol for this systematic review has been registered in the Research Registry 2020 under number reviewregistry923**.**

### 2.2 Eligible criteria for study selection

#### 2.2.1 Types of studies

Only RCTs investigating herbal medicines for the prevention or treatment of CVS after SAH will be included in this study, without publication or language restriction. Non-RCTs, case reports, case series, uncontrolled trials, and laboratory studies will be excluded, as will trials that fail to provide detailed results.

#### 2.2.2 Types of participants

Eligible participants will be defined as patients with SAH or CVS after SAH. Brain imaging, including computed tomography, magnetic resonance imaging, and transcranial Doppler, will be used to diagnose SAH or CVS after SAH. There will be no restrictions based on gender, ethnicity, symptom severity, disease duration, and clinical setting. However, patients with other cerebral hemorrhagic conditions, such as subdural hemorrhage and intracerebral hemorrhage, will be excluded.

#### 2.2.3 Types of interventions

We will include studies using adjunct herbal medicines + conventional Western medicine therapies (including surgery) as experimental interventions, with no limitations on dosage, frequency, duration of treatment, and formulation, for example, decoctions, extracts, tablets, capsules, and powders. The present study only included oral administration of herbal medicines. Therefore, intravenous or acupuncture point injections of herbal medicines will be excluded.

The control interventions will include placebo + conventional Western medicine therapies (including surgery), or conventional Western medicine therapies (including surgery) only. We will exclude studies comparing other traditional Chinese medicine therapies, (including traditional Korean medicine and Japanese Kampo medicine), such as those using different types of herbal medicines. Studies comparing the effect of herbal medicines with acupuncture treatment or moxibustion will also be excluded.

#### 2.2.4 Types of outcome measures

For the primary outcome, we will assess the prevalence or recurrence of CVS. For secondary outcomes, we will include other parameters, such as the prevalence of vasospasm related cerebral infarction, National Institute of Health Stroke Scale (NIHSS), modified Rankin Scale (mRS), recurrence rate of SAH, and prevalence of other complications, such as hydrocephalus. We will also investigate the number and severity of adverse events.

### 2.3 Search methods for the identification of studies

#### 2.3.1 Electronic searches

The following databases will be searched from inception to June 2020: MEDLINE (via PubMed), the Cochrane Central Register of Controlled Trials (CENTRAL), Scopus, Citation Information by Nii (CiNii), China National Knowledge Infrastructure Database (CNKI), Oriental Medicine Advanced Searching Integrated System (OASIS), and National Digital Science Library (NDSL). The specific search strategies (for example, for PubMed) are shown in Table 1. We will make relative modifications in accordance with the requirements, and an equivalent translation of the search terms will be adopted to ensure that similar search terms are used in all databases. If we need additional information for the identified studies, we will contact the corresponding authors.

**Table 1:** Search strategy for PubMed.

#1	Search “Subarachnoid hemorrhage” [Mesh]
#2	Search “subarachnoid hem^∗^” [tiab]
#3	Search “Vasospasm, Intracranial” [Mesh]
#4	Search “cerebral vasospasm^∗^” [tiab]
#5	#1 or #2 or #3 or #4
#6	Search (“herbal med^∗^”[tiab] OR “herbal com^∗^”[tiab] OR “herb^∗^”[tiab])
#7	Search (“traditional kor^∗^”[tiab] OR “korean med^∗^”[tiab])
#8	Search “traditional chin^∗^”[tiab]
#9	Search (“kanpo”[tiab] OR “kampo”[tiab])
#10	Search “decoction ”[tiab]
#11	#6 or #7 or #8 or #9 or #10
#12	Search (“randomise^∗^”[tiab] OR “randomize^∗^”[tiab])
#13	#5 and #11 and # 12
#14	Search (“animals” [Mesh] NOT “humans” [Mesh])
#15	#13 NOT #14

#### 2.3.2 Search for other resources

A manual search will also be performed to search the reference lists of the relevant articles.

### 2.4 Data collection and analysis

#### 2.4.1 Study selection

**Figure 1:**
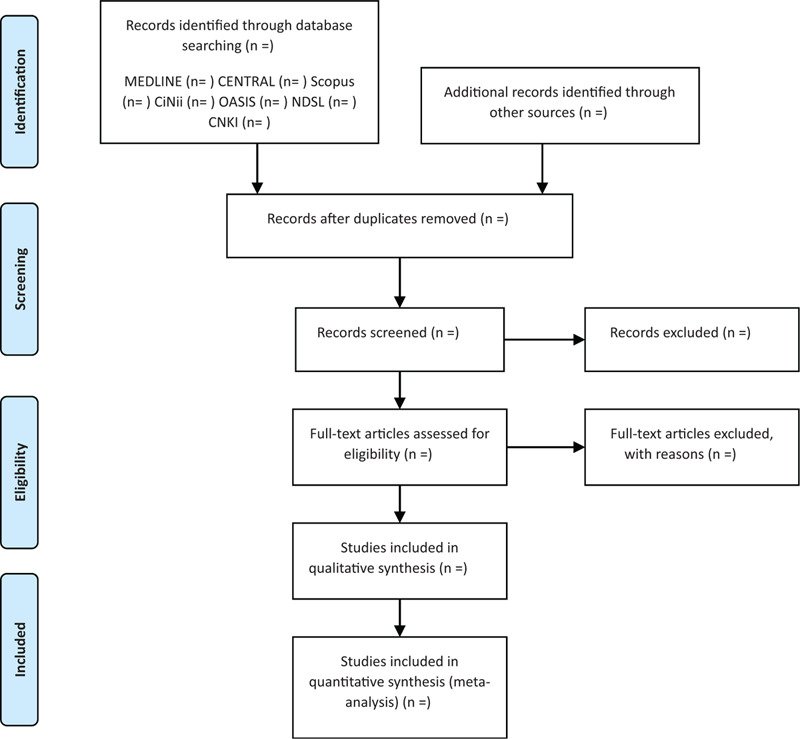
A PRISMA flow diagram of literature screening and selection processes.

Two reviewers (SK and CJ) who have been trained on the process and purpose of selection will independently review the titles, abstracts, and manuscripts of the studies and screen them for eligibility for inclusion in the analysis. After removing duplicates, full texts were reviewed. All studies, identified by both electronic and manual searches, will be uploaded to EndNote X7 (Clarivate Analytics), and the reasons for excluding studies will be recorded and shown in a PRISMA flowchart, as shown in Figure 1. All disagreements will be resolved by consensus and discussion between the 2 reviewers.

#### 2.4.2 Data extraction and management

Two review writers (SK and CJ) will independently extract the data and fill out the standard data extraction form, which includes study information such as the first author, publication year, language, sample size, characteristics of participants (e.g., age and sex), details of randomization, blinding, interventions, treatment period, outcome measures, primary outcome, secondary outcome, and statistical method used. If there are disagreements, another review writer (SY) will be called in to help make the decision.

#### 2.4.3 Assessment of bias risk and quality of included studies

Two reviewers (SK and CJ) will assess the risk of bias (RoB) based on the Cochrane Collaboration's tool,^[17]^ which includes references to the following: random sequence generation (selection bias), allocation concealment (selection bias), blinding of participants and personnel (performance bias), blinding of outcome assessment data (detection bias), incomplete outcome data (attribution bias), selective reporting (reporting bias), and other bias. The assessment results will be shown as one of the 3 categories: low, unclear, and high.

#### 2.4.4 Measurement of treatment effect

For continuous data, the pooled results will be presented as the mean difference (MD) or standardized MD with 95% confidence intervals (CIs). For dichotomous data, the pooled results will be presented as a risk ratio (RR) with 95% CIs.

#### 2.4.5 Managing missing data

If there are any missing, insufficient, or unclear data, we will contact the corresponding author and gather the relevant information. If the information cannot be obtained, only the remaining available information will be analyzed, which will be discussed.

#### 2.4.6 Assessment of heterogeneity

We will perform the *I*^2^ test to evaluate statistical heterogeneity. If the *I*^2^ will be greater than 50%, statistical heterogeneity will be considered.

#### 2.4.7 Data synthesis

We will use the Review Manager program (V.5.3.5 Copenhagen: The Nordic Cochrane Center. The Cochrane Collaboration, 2014) for statistical analysis. If *I*^2^ ≤ 50%, the fixed-effect model will be employed to evaluate the outcome data. Otherwise, a random-effects model will be applied. The studies will be synthesized according to the type of intervention and/or control as follows:

1. Herbal medicines + conventional Western medicine therapies (including surgery) vs only conventional Western medicine therapies (including surgery)
2. Herbal medicines + conventional Western medicine therapies (including surgery) vs placebo + conventional Western medicine therapies (including surgery).

The heterogeneity levels will be assessed in the included literature, and if enough studies are available to investigate the causes of heterogeneity and its criteria, the groups indicated below ('Subgroup analysis section’) will be assessed. We will use the Grading of Recommendations Assessment, Development and Evaluation (GRADE) pro software from Cochrane Systematic Reviews to create a Summary of Findings table.

#### 2.4.8 Subgroup analysis

If sufficient studies are available to investigate the cause of heterogeneity and its criteria, the following will be assessed: the form of the herbal medicine such as granules or decoctions, the name of the herbal medicine used, the types of surgery, and the types of diagnosis, such as SAH without CVS or SAH with CVS.

#### 2.4.9 Sensitivity analysis

We will perform a sensitivity analysis to verify the robustness of the study results. This will be done by assessing the impact of sample size, high RoB, missing data, and selected models. Following the analyses, if the quality of the studies is judged to be low, these studies will be removed to ensure the robustness of the results.

#### 2.4.10 Ethics and dissemination

Formal ethical approval is not required in this protocol. We will collect and analyze data based on published studies, and since no patients are directly or specifically assessed in this study, individual privacy will not be a concern. The results of this review will be disseminated to peer-reviewed journals or presented at a relevant conference.

## 3 Discussion

CVS is a major complication that worsens the prognosis of patients with SAH.^[1,2]^ CVS is one of the leading causes of death in patients with SA.^[1,2]^ Even if they survive, it causes DIND and long-term disability.^[7]^ As a result, the quality of life of patients with SAH is significantly lowered,^[7]^ and an economic burden is caused by continuous rehabilitation treatment.^[18]^ However, there is still no definite intervention for CVS prevention and treatment.

According to previous studies, various herbs can have beneficial effects on CVS through anti-inflammatory, anti-edema, antioxidant, anti-platelet, and anti-leukocyte aggregation effects, as well as effects on vascular endothelial cells.^[14]^ In addition, some clinical trials suggest that herbal complexes such as Angong Niuhuang Pills,^[19]^ Boyanghwano-tang,^[15]^ and Qingnao Oral Liquid^[15]^ could be effective in treating CVS or headache after SAH. Thus, herbal medicines are likely to be new alternatives to prevent and treat CVS after SAH, which may have been a problem for the prognosis of patients with SAH.

The present review will be conducted to assess the efficacy and safety of using herbal medicine to prevent and treat CVS after SAH, to establish novel management strategies that reduce the burden of practitioners, patients, and their families.

## Author contributions

**Conceptualization:** Yuna Seo, Seungwon Kwon.

**Data curation:** Han-Gyul Lee, Chul Jin, Seung-bo Yang, Seung-Yeon Cho, Woo-Sang Jung.

**Formal analysis:** Han-Gyul Lee, Seung-bo Yang, Seong-Uk Park, Woo-Sang Jung, Sang-Kwan Moon, Jung-Mi Park.

**Funding acquisition:** Seungwon Kwon.

**Project administration:** Seungwon Kwon.

**Writing – original draft:** Yuna Seo, Seungwon Kwon.

**Writing – review & editing:** Seung-bo Yang, Chang-Nam Ko, Ki-Ho Cho, Seungwon Kwon.
